# Recent TIPS increases postoperative mortality: A national cohort study

**DOI:** 10.1097/HC9.0000000000000577

**Published:** 2024-11-29

**Authors:** Helen Tang, David E. Kaplan, Samir Abu-Gazala, Nadim Mahmud

**Affiliations:** 1 Department of Medicine, Perelman School of Medicine, University of Pennsylvania, Philadelphia, Pennsylvania, USA; 2 Department of Medicine, Division of Gastroenterology and Hepatology, Perelman School of Medicine, University of Pennsylvania, Philadelphia, Pennsylvania, USA; 3 Department of Medicine, Corporal Michael J. Crescenz VA Medical Center, Philadelphia, Pennsylvania, USA; 4 Department of Surgery, Division of Transplant Surgery, Perelman School of Medicine, University of Pennsylvania, Philadelphia, Pennsylvania, USA; 5 Leonard Davis Institute of Health Economics, University of Pennsylvania, Philadelphia, Pennsylvania, USA; 6 Department of Biostatistics, Epidemiology & Informatics, Center for Clinical Epidemiology and Biostatistics, Perelman School of Medicine, University of Pennsylvania, Philadelphia, Pennsylvania, USA

**Keywords:** cirrhosis, liver function, portal hypertension, surgical risk, TIPS

## Abstract

**Background:**

Patients with cirrhosis have an increased risk of postoperative mortality, which is partially attributable to portal hypertension. Preoperative TIPS placement may reduce operative risk. Studies suggesting the benefits of preoperative TIPS are limited by residual confounding and lack of longitudinal laboratory data. To address these limitations, we used granular longitudinal data from the Veterans Health Administration.

**Methods:**

This retrospective cohort study of Veterans Health Administration patients with cirrhosis who underwent major surgery from 2008 to 2022 identified patients who underwent TIPS placement within 6 months before surgery. Demographics, comorbidities, surgery type, and longitudinal laboratory data were incorporated into a propensity score using 5:1 caliper matching for receipt of TIPS. The propensity-matched cohort included 39 patients with preoperative TIPS and 171 without.

**Results:**

Baseline characteristics were similar between groups. In Cox regression, recent TIPS was associated with an increased risk of postoperative mortality (HR: 2.69, 95% CI: 1.37–5.30, *p* = 0.004), redemonstrated in 500 random resampling events (median HR: 1.71). TIPS and non-TIPS patients had similar albumin, bilirubin, and international normalized ratio 6 months before surgery; however, immediately before surgery, TIPS patients had lower albumin (*p* = 0.009), higher bilirubin (*p* = 0.001), and higher international normalized ratio (*p* = 0.001).

**Conclusions:**

In a propensity-matched analysis of patients with cirrhosis undergoing major surgery, recent TIPS was associated with increased postoperative mortality and worsened liver synthetic function in the immediate preoperative period. TIPS placement should be carefully considered in patients with cirrhosis who may undergo surgery.

## INTRODUCTION

Patients with cirrhosis undergoing major surgery have an increased risk of postoperative mortality.^1^ This is attributable to factors including poor liver synthetic function, sarcopenia, frailty, relative coagulopathy, and portal hypertension, thus spurring the development of risk prediction models for postoperative decompensation and mortality.^2^ Portal hypertension is a particularly ominous risk factor, with increased HVPG increasing the risk of intraoperative bleeding, impaired hemostasis, and postoperative decompensation and mortality.^3,4^ TIPS, typically used in refractory ascites or as secondary prevention after variceal bleeding, directly decreases HVPG. Thus, preoperative TIPS placement may mitigate the risks of portal hypertension and improve postoperative outcomes in selected patients with cirrhosis.

Though the theoretical benefit of preoperative TIPS was first suggested decades ago, the literature remains conflicting, and TIPS is not without its own risks of HE and right heart overload. In the initial uncontrolled case series, preoperative TIPS was associated with acceptable rates of short-term morbidity, mortality, and TIPS complications.^5,6,7^ Early comparative studies demonstrated an unchanged risk of postoperative mortality in major abdominal surgeries preceded by preoperative TIPS.^8,9,10^ More recently, retrospective cohort studies with matched or adjusted analysis have demonstrated no difference^11,12^ or potential reduction in postoperative mortality in patients with preoperative TIPS.^13,14,15^ No prospective or randomized studies have been performed, and thus retrospective studies are limited by heterogeneous indications for TIPS, with a minority placed specifically for preoperative risk mitigation. Other limitations of prior retrospective studies with matched control cohorts include small sample sizes, residual confounding, exclusive focus on postoperative mortality, and limited exploration of trends in laboratory or clinical data. In particular, adjustment or matching based on immediate preoperative labs could result in biased estimates of effect given that TIPS may plausibly lead to worsened liver synthetic function in the interval between TIPS placement and surgery. To address these limitations, we used granular data from the Veterans Health Administration (VHA) to explore the association between recently placed TIPS and postoperative mortality, with special consideration of longitudinal laboratory changes in the pre-TIPS baseline, immediate preoperative, and postoperative periods.

## METHODS

### Study design and cohort creation

This retrospective cohort study used data from an updated Veterans Outcomes and Costs Associated with Liver Diseases (VOCAL) cohort merged with the Veterans Affairs Surgical Quality Improvement Program (VASQIP) data set, representing 110 VHA centers across the United States. VOCAL-VASQIP is a well-established cohort of patients with cirrhosis, identified using a validated VHA algorithm,^16^ who underwent surgical procedures between 2008 and 2022. VASQIP contains manually adjudicated data on surgical procedures, including preoperative, intraoperative, and postoperative outcomes data.^2^ We included patients with cirrhosis who underwent a major nonhepatic surgery of interest (detailed below) and excluded patients with prior liver transplantation.

### Exposures and outcomes

The primary exposure of interest was the presence of TIPS placed in a recent preoperative time period, defined as placement of TIPS within 6 months before major surgery, regardless of the indication for placement. Current Procedure Terminology (CPT) codes were used to identify TIPS procedures performed both within and outside the VHA system (through Fee Basis tables), and patient charts were manually adjudicated to determine the reported indication for TIPS placement. We extracted detailed demographic and comorbidity data using previously published methods,^17,18^ and etiology of liver disease was classified using a validated VHA algorithm.^19^ CPT codes were used to categorize surgeries into spine/central nervous system, rectum/genitourinary/retroperitoneal, orthopedic, abdominal wall, vascular, chest/cardiac, or major abdominal (Supplemental Table S1, http://links.lww.com/HC9/B88).^2,20^ American Society of Anesthesiologists (ASA) physical status classification and emergent versus elective surgery status were obtained from preoperative anesthesiology documentation. Longitudinal laboratory data were extracted at 3 time points: (1) baseline period, defined as the closest labs before the 6-month marker before surgery, (2) preoperative, defined as labs obtained immediately preceding surgery, and (3) postoperative labs through 28 days. The Model for End-Stage Liver Disease-Sodium (MELD-Na) was computed from laboratory values. Finally, the VOCAL-Penn Score (VPS) predictions for 30-, 90-, and 180-day postoperative mortality were calculated separately using baseline period labs and immediate preoperative labs.

The primary outcome was time to postoperative mortality, assessed through 12 months of follow-up. Secondary outcomes included intraoperative blood loss, surgery duration, and postoperative length of stay. Exploratory outcomes included trajectories of laboratory studies relevant to liver function over time.

### Statistical analysis

Descriptive statistics were reported as frequencies and percentages for categorical variables and as medians and IQRs for continuous variables. Statistical comparisons between groups were performed using Wilcoxon rank-sum and chi-square tests for continuous and categorical data, respectively. To balance baseline covariates between preoperative TIPS and control groups, we used propensity score matching (PSM). Using baseline data from 6 months before surgery, we fit a logistic regression model with preoperative TIPS as the outcome with the following covariates: age, sex, race/ethnicity, liver disease etiology, coronary artery disease, heart failure, ascites, HE, total bilirubin, albumin, sodium, hemoglobin, surgery type, ASA score, and emergency versus elective surgery. A propensity score was generated from this model and 5:1 controls:cases nearest neighbor matching with replacement (caliper width 0.001) was used to identify the analytic cohort. Box plots of propensity scores were created for unmatched and matched cohorts, and descriptive data for all available variables in the analytic cohort were computed to demonstrate the adequacy of matching.

To evaluate the association between preoperative TIPS and all-cause postoperative mortality, we plotted Kaplan-Meier curves, which were compared using stratified log-rank tests given the lack of independent observations due to PSM. We estimated the HR and 95% CI using univariable Cox regression, again accounting for matching. Time zero was the day of surgery. Data were right-censored at maximum follow-up. Competing risk analysis was not performed as only 2 transplant events occurred, both near the end of follow-up (361 and 362 d after surgery in the non-TIPS group). Finally, given the large pool of potential controls, we repeated the above process 500 times to assess the variability in HR estimates with repeated random number generator PSM iterations. HRs were plotted using a histogram, and the median HR and IQR were reported.

### Sensitivity analysis

Given the possibility that TIPS in the 3–6 months before surgery may not have been performed with the primary purpose of minimizing operative risk, we performed a sensitivity analysis where we isolated TIPS placed in the 0–3 months before surgery and performed propensity matching using baseline laboratory and comorbid data at the 3-month time point before surgery, more closely simulating the timeframe used in prior studies. Kaplan-Meier and Cox regression analyses were then performed similar to the primary analysis.

### Exploratory analyses

Trajectories of key liver-related labs (albumin, total bilirubin, international normalized ratio [INR], and platelet count) were plotted using bar graphs between preoperative TIPS and control groups in each of the 3 time points detailed previously: baseline period (6 mo before surgery), immediate preoperative period (closest labs before surgery), and postoperative period through 28 days. In the latter group, minimum values for albumin and platelet count, and maximum values for total bilirubin and INR through postoperative day 28 were used. Comparisons were made using Wilcoxon rank-sum tests. Next, to identify laboratory phenotypes of patients with different outcome trajectories, descriptive statistics for longitudinal laboratory data were compared among patients who survived or died, stratified by preoperative TIPS status. Median predicted probabilities and IQRs of postoperative mortality from the VPS were presented for each group (preoperative TIPS or no preoperative TIPS, survival or death). C-statistics were also presented, with values >0.7 representing good discrimination and >0.8 representing excellent discrimination.

Finally, to better understand potential risk factors and mechanisms impacting perioperative risk in the context of TIPS placement, we performed manual adjudication of all patient charts (Helen Tang) in the primary PSM cohort to determine preoperative TIPS imaging, TIPS patency, presence/absence of preoperative ascites, cancer-related surgical indication, and postoperative cause of death (liver-related, cardiovascular-related, infection-related, and other/unknown). We also determined specific surgical indications for incarcerated abdominal hernia, which may be a complication of TIPS placement resulting in the need for surgery.

### Other considerations

This study was conducted in accordance with the Declarations of Helsinki and Istanbul and received IRB approval from the Michael J. Crescenz Philadelphia Veterans Affairs Medical Center. All analyses were performed using STATA 18.0/BE.

## RESULTS

### Cohort characteristics

After PSM, 210 patients were included in the analytic cohort: 39 who underwent TIPS in the preoperative period and 171 who did not (Table 1). Prematching characteristics, including 13,439 potential no-TIPS controls, are shown in Supplemental Table S2, http://links.lww.com/HC9/B88. After matching, there were no statistically significant differences between groups in baseline characteristics; the distribution of propensity scores was also similar (Supplemental Figure S1, http://links.lww.com/HC9/B88). Most patients were male, with alcohol-associated liver disease as the most common etiology of liver disease. In the TIPS group, the median interval from TIPS placement to surgery was 56 days (IQR: 22, 105). Primary indications for TIPS placement were refractory ascites (n = 22, 56.4%), preoperative risk mitigation (n = 8, 20.5%), variceal hemorrhage (n = 5, 12.8%), and unknown (n = 4, 10.3%).

**TABLE 1:** Cohort characteristics after propensity score matching

Factor	No preoperative TIPS (N = 171)	Preoperative TIPS (N = 39)	*p*
Age, median (IQR)	63 (57, 68)	63 (55, 68)	0.65
Sex, male, n (%)	168 (98.2)	38 (97.4)	0.74
Race/ethnicity, n (%)			0.85
White	135 (78.9)	32 (82.1)	
Black	6 (3.5)	1 (2.6)	
Hispanic	10 (5.8)	1 (2.6)	
Other	20 (11.7)	5 (12.8)	
Etiology, n (%)			0.99
Hepatitis C virus	16 (9.4)	4 (10.3)	
Alcohol-associated liver disease	100 (58.5)	21 (53.8)	
HCV + ALD	24 (14.0)	6 (15.4)	
MASLD	27 (15.8)	7 (17.9)	
Other	4 (2.3)	1 (2.6)	
Type of surgery, n (%)			0.91
Spine/CNS	8 (4.7)	1 (2.6)	
Rectum/GU/RP	7 (4.1)	2 (5.1)	
Ortho	30 (17.5)	5 (12.8)	
Abdominal wall	83 (48.5)	21 (53.8)	
Major abdominal	43 (25.1)	10 (25.6)	
ASA classification, n (%)			0.95
2	7 (4.1)	1 (2.6)	
3	90 (52.6)	20 (51.3)	
4	71 (41.5)	17 (43.6)	
5	3 (1.8)	1 (2.6)	
Emergency status, n (%)	50 (29.2)	13 (33.3)	0.61
Baseline comorbidities (6 mo before surgery), n (%)			
Diabetes mellitus	102 (59.6)	23 (59.0)	0.94
Coronary artery disease	42 (24.6)	10 (25.6)	0.89
Heart failure	15 (8.8)	3 (7.7)	0.83
Ascites	122 (71.3)	31 (79.5)	0.30
HE	42 (24.6)	10 (25.6)	0.89
Baseline laboratory studies (6 mo before surgery)			
MELD-Na, median (IQR)	14 (10, 19)	13 (10, 16.5)	0.63
Creatinine, median (IQR)	1.0 (0.8, 1.3)	1.0 (0.8, 1.6)	0.97
Sodium, median (IQR)	137 (134, 139)	137 (134, 139)	0.53
Albumin, median (IQR)	3.4 (2.9, 3.8)	3.1 (2.8, 3.9)	0.39
Bilirubin, median (IQR)	0.9 (0.6, 1.7)	1.2 (0.7, 1.7)	0.15
INR, median (IQR)	1.2 (1.1, 1.4)	1.2 (1.1, 1.3)	0.46
Hemoglobin, median (IQR)	11.7 (10.2, 13.6)	11.7 (10.2, 13.6)	0.92
Platelet count, median (IQR)	143 (100, 200)	152 (85, 217)	0.73

Abbreviations: ALD, alcohol-associated liver disease; ASA, American Society of Anesthesiologists; CNS, central nervous system; GU, genitourinary; INR, international normalized ratio; MASLD, metabolic dysfunction–associated steatotic liver disease; MELD-Na, Model for End-Stage Liver Disease-Sodium; RP, retroperitoneal.

### Operative outcomes

**FIGURE 1:**
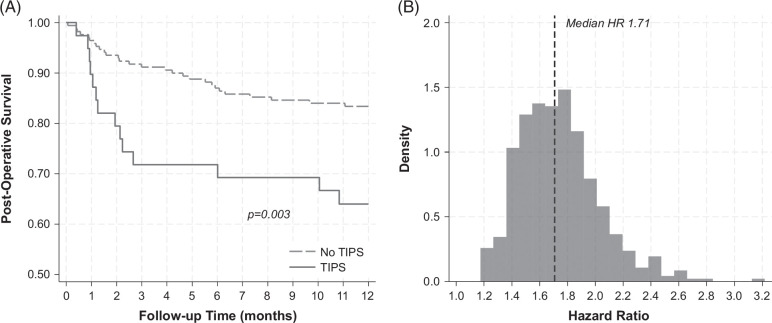
(A) Kaplan-Meier analysis of the association between preoperative TIPS and postoperative mortality and (B) resampling estimates for hazard ratio in 500 random propensity-matched samples.

Over a median of 12.0 months (IQR: 11.1, 12.0), a total of 14 (35.9%) TIPS patients died compared to 28 (16.4%) patients without TIPS. In the overall PSM cohort, the highest mortality rates were observed in rectum/genitourinary/retroperitoneal surgeries (33.3%), major abdominal (22.6%), and major orthopedic surgeries (22.6%), and the lowest in spine/central nervous system surgeries (12.5%). On Kaplan-Meier analysis, there were significant differences in survival distributions between TIPS and no-TIPS patients (Figure 1A; stratified log-rank *p* = 0.003). Cumulative mortality incidences are summarized in Table 2; for example, 1-month mortality was 10.26% versus 3.53% in TIPS versus no-TIPS groups, respectively, and 12-month mortality was 36.00% versus 16.65%. In Cox regression accounting for matching, patients who underwent preoperative TIPS had a 2.69-fold increased hazard for postoperative mortality relative to those without preoperative TIPS (HR: 2.69, 95% CI: 1.37–5.30, *p* = 0.004). This finding was redemonstrated when PSM and analyses were repeated 500 times using a random number generator, where all HR parameter estimates were >1 and the median HR was 1.71 (Figure 1B). Finally, in a sensitivity analysis where analyses were repeated when limiting preoperative TIPS procedures identified in the 3 months before surgery, a total of 27 patients who received TIPS were matched to 133 no-TIPS controls with an excellent balance of propensity scores (Supplemental Figure S2, http://links.lww.com/HC9/B88). In Kaplan-Meier analysis, there remained significant differences in postoperative mortality between groups (log-rank *p* = 0.02; Supplemental Figure S3, http://links.lww.com/HC9/B88), and Cox regression demonstrated a 2.74-fold increased hazard of postoperative mortality associated with preoperative TIPS placement (HR: 2.74, 95% CI: 1.13–6.61, *p* = 0.025).

**TABLE 2:** Cumulative incidence of postoperative mortality for preoperative TIPS and no preoperative TIPS groups

Postoperative time point (mo)	No preoperative TIPS (N = 171)	Preoperative TIPS (N = 39)
1	0.0353	0.1026
3	0.0882	0.2821
6	0.1300	0.2821
12	0.1665	0.3600

Regarding secondary outcomes, there were no significant differences in reported intraoperative blood loss between preoperative TIPS and no preoperative TIPS groups (median: 50 mL [IQR: 10, 100] vs. 25 mL [IQR: 10, 100], respectively, *p* = 0.63), no differences in surgery duration (median: 98 min [IQR 69, 127] vs. 92 min [IQR: 55, 135], *p* = 0.24), and no differences in postoperative length of stay (median: 7 d [IQR: 5, 12] vs. 5 d [IQR: 2, 9], *p* = 0.07).

### Trajectory of liver function–related laboratory values

**FIGURE 2:**
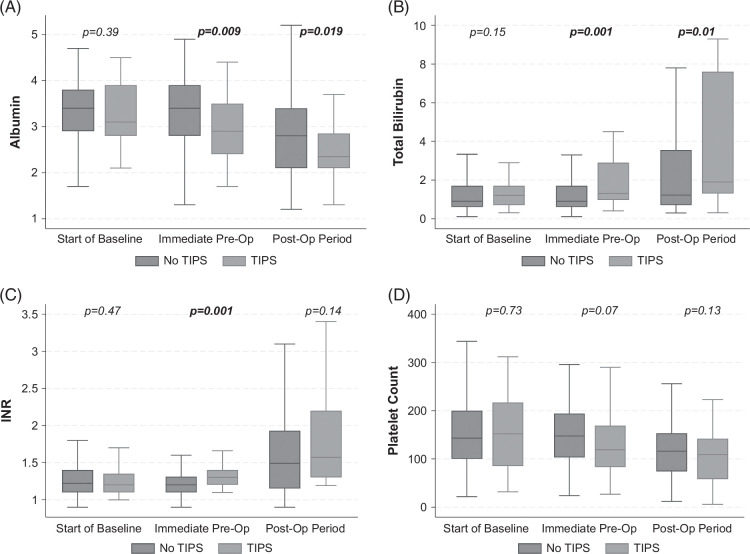
Trajectories in liver-related labs across baseline, preoperative, and postoperative periods for (A) albumin, (B) total bilirubin, (C) international normalized ratio, and (D) platelet count for preoperative TIPS and no preoperative TIPS groups *Start of baseline refers to labs at 6 months before surgery. Immediate pre-op labs are those closest to the operative date before surgery. Postoperative labs are maximums (total bilirubin and INR) or minimums (albumin and platelet count) through 28 days after surgery. p values in bold are statistically significant at the 5% alpha level.* Abbreviation: INR, international normalized ratio

At a 6-month preoperative baseline, there were no differences in albumin, total bilirubin, INR, or platelet count between preoperative TIPS and no preoperative TIPS groups (Figures 2A–D). In the immediate preoperative period, prior TIPS was associated with lower albumin (*p* = 0.009), higher total bilirubin (*p* = 0.001), and higher INR (*p* = 0.001) compared to matched controls with no preoperative TIPS. Postoperatively, the TIPS group had lower albumin (*p* = 0.019) and higher total bilirubin (*p* = 0.01) than controls. Platelet count was not statistically different between groups preoperatively (*p* = 0.07) or postoperatively (*p* = 0.13).

In exploratory analysis, laboratory values were compared between patients who died postoperatively with and without preoperative TIPS. For example, while there were no statistically significant differences in albumin, INR, platelet count, hemoglobin, or sodium at baseline, there were significant differences between groups in most preoperative and postoperative labs (Table 3). In the immediate preoperative period, patients who would ultimately die in the postoperative period had lower albumin, lower hemoglobin, higher bilirubin, higher INR, and higher MELD-Na relative to postoperative survivors (each *p* < 0.05). In the postoperative period, post-TIPS nonsurvivors had the most extreme liver-related laboratory findings, even relative to nonsurvivors without TIPS. This included notably higher bilirubin (median 7.9 vs. 3.4, *p* < 0.001) and higher MELD-Na (median 30 vs. 25, *p* = 0.001).

**TABLE 3:** Descriptive data of laboratory trajectories for preoperative versus no preoperative TIPS, stratified by survival status through 12 months

	No preoperative TIPS (N = 171)		Preoperative TIPS (N = 39)		
	Survival (n = 143)	Death (n = 28)	Survival (n = 25)	Death (n = 14)	*p*
Baseline period^a^					
Albumin, median (IQR)	3.5 (2.9, 3.8)	3.0 (2.7, 3.4)	3.1 (2.8, 3.9)	3.2 (2.9, 3.8)	0.05
Bilirubin, median (IQR)	0.8 (0.6, 1.5)	1.7 (0.8, 2.7)	1.3 (0.7, 1.7)	1.0 (0.8, 1.5)	**0.02**
INR, median (IQR)	1.2 (1.1, 1.4)	1.3 (1.2, 1.4)	1.2 (1.1, 1.5)	1.2 (1.1, 1.2)	0.48
Platelets, median (IQR)	146 (102, 206)	114 (89.5, 175)	160 (95, 217)	123.5 (85, 174)	0.44
Hemoglobin, median (IQR)	11.9 (10.3, 13.6)	11.1 (10.1, 13.0)	11.7 (10.2, 13.4)	11.6 (10.6, 13.6)	0.69
Creatinine, median (IQR)	1.0 (0.8, 1.3)	1.1 (1.0, 2.2)	1.0 (0.8, 1.5)	1.0 (0.9, 1.7)	0.43
Sodium, median (IQR)	137 (134, 139)	136 (133, 138)	137 (134, 139)	137 (136, 138)	0.76
MELD-Na, median (IQR)	13 (10, 19)	16 (13, 22)	13 (12, 18)	13 (9, 16)	**0.048**
Preoperative period					
Albumin, median (IQR)	3.5 (3.1, 4.0)	2.7 (2.1, 3.5)	3.0 (2.4, 3.5)	2.7 (2.4, 3.4)	**<0.001**
Bilirubin, median (IQR)	0.9 (0.6, 1.4)	1.8 (0.9, 2.9)	1.2 (0.9, 2.1)	2.2 (1.1, 6.1)	**<0.001**
INR, median (IQR)	1.1 (1.1, 1.3)	1.3 (1.2, 1.5)	1.3 (1.2, 1.3)	1.4 (1.3, 1.7)	**<0.001**
Platelets, median (IQR)	148 (107, 195)	122 (91.5, 192.5)	135 (97, 170)	112.5 (50, 157)	0.11
Hemoglobin, median (IQR)	11.9 (10.7, 13.5)	10.1 (8.4, 11.5)	9.8 (9.3, 11.6)	9.4 (8.8, 11.7)	**<0.001**
Creatinine, median (IQR)	1.1 (0.9, 1.5)	1.2 (0.8, 2.1)	0.9 (0.8, 1.3)	1.0 (0.7, 1.4)	0.70
Sodium, median (IQR)	137 (135, 139)	136 (130, 139)	137 (135, 140)	136 (135, 139)	0.60
MELD-Na, median (IQR)	13 (10, 18)	17.5 (13.5, 23.5)	15 (11, 18)	16.5 (14, 22)	**0.002**
Postoperative period					
Albumin, median (IQR)	3.0 (2.4, 3.4)	2.2 (1.7, 2.7)	2.4 (2.1, 2.9)	2.3 (1.9, 2.7)	**<0.001**
Bilirubin, median (IQR)	1.1 (0.7, 2.3)	3.4 (0.8, 6.9)	1.7 (1.0, 1.9)	7.9 (2.0, 21.8)	**<0.001**
INR, median (IQR)	1.4 (1.1, 1.8)	1.6 (1.3, 2.0)	1.4 (1.2, 1.8)	1.7 (1.5, 2.7)	**0.024**
Platelets, median (IQR)	124 (82, 161)	88.5 (57, 130)	126 (60, 169)	72.5 (20, 111)	**0.004**
Hemoglobin, median (IQR)	9.6 (7.9, 12.0)	7.6 (6.8, 8.6)	8.1 (6.9, 9.1)	8.2 (6.5, 10.4)	**<0.001**
Creatinine, median (IQR)	1.2 (0.9, 2.0)	2.1 (1.4, 3.2)	1.1 (0.9, 1.8)	1.7 (1.0, 2.7)	**0.019**
Sodium, median (IQR)	133 (130, 136)	130 (127, 133)	133 (131, 137)	133 (131, 133)	**0.017**
MELD-Na, median (IQR)	18 (14, 26)	25 (19, 32)	17 (15, 23)	30 (27, 36)	**0.001**

^a^ Baseline period corresponds to 6 months before surgery.

Abbreviations: INR, international normalized ratio; MELD-Na, Model for End-Stage Liver Disease-Sodium

### Application of the VOCAL-Penn surgical risk score

Using both 6-month baseline and immediate preoperative laboratory values, the VPS was calculated to predict 30-, 90-, and 180-day mortality (Table 4). Discrimination of the model for patients without preoperative TIPS was very good to excellent when baseline labs were used (C-statistics: 0.774–0.836) and uniformly excellent when preoperative labs were used (C-statistics: 0.818–0.875). In the TIPS group, discrimination was very good when baseline labs were used (C-statistics: 0.733–0.794), but very good to excellent when immediate preoperative labs were used (C-statistics: 0.733–0.892). Despite higher postoperative mortality for preoperative TIPS patients, the VPS predicted a slightly lower postoperative risk in these patients relative to patients without preoperative TIPS, indicating an underestimation of surgical risk in patients with preoperative TIPS.

**TABLE 4:** VOCAL-Penn Score prediction distributions (%) and C-statistics for preoperative TIPS and no preoperative TIPS groups, computed using 6-month baseline labs and immediate preoperative labs

	No preoperative TIPS (N = 171)			Preoperative TIPS (N = 39)		
	Survival	Death	C-statistic	Survival	Death	C-statistic
Baseline labs^a^						
30-d mortality	2.3 (0.8, 5.3)	8.7 (4.8, 12.5)	0.836	2.8 (1.5, 5.5)	4.6 (3.6, 6.5)	0.794
90-d mortality	4.4 (1.9, 11.5)	13.5 (9.4, 23.3)	0.806	5.0 (2.6, 9.7)	8.0 (7.3, 11.5)	0.733
180-d mortality	7.0 (3.3, 15.7)	17.7 (12.7, 30.2)	0.774	7.5 (4.2, 13.4)	13.5 (9.8, 16.1)	0.741
Preoperative labs						
30-d mortality	2.0 (0.7, 6.7)	10.9 (4.9, 15.8)	0.875	4.1 (1.7, 8.5)	8.5 (4.7, 14.6)	0.892
90-d mortality	4.0 (1.5, 11.4)	18.9 (10.8, 26.3)	0.818	7.7 (3.6, 12.7)	13.6 (8.2, 28.1)	0.733
180-d mortality	6.4 (2.8, 16.4)	24.5 (17.5, 31.6)	0.825	10.3 (5.7, 20.4)	18.7 (12.7, 34.5)	0.759

^a^ VOCAL-Penn Score predictions computed using laboratory data 6 months before surgery.

Abbreviation: VOCAL, Veterans Outcomes and Costs Associated with Liver Diseases.

### Exploratory analysis of chart-adjudicated data

After a manual review of all primary PSM patient medical records, there was no significant difference in cancer-related surgical indications between TIPS and no-TIPS patients (12.8% vs. 6.4%, *p* = 0.17). Preoperative TIPS imaging (84% ultrasound and 16% CT) was performed in 79.5% of TIPS patients, of which TIPS patency was demonstrated in all but 1 case (96.8%). By exam or imaging, 13 (33%) of TIPS patients had some degree of ascites before surgery. Among patients who underwent abdominal wall surgery, incarceration of abdominal hernia was significantly more common as a surgical indication in TIPS versus no-TIPS patients (71% vs. 5%, *p* < 0.001); all of these occurred in patients with ascites at baseline. Finally, among patients who experienced postoperative mortality through 1 year (n = 14 for TIPS and n = 28 for no TIPS), there were no significant differences in causes of death between TIPS and no-TIPS patients (*p* = 0.64); these included infection-related death (14% TIPS vs. 14% no TIPS), cardiovascular-related death (7% TIPS vs. 7% no TIPS), liver-related death (14% vs. 32%), and other/unknown (64% vs. 46%).

## DISCUSSION

In this retrospective study of VHA patients with cirrhosis undergoing surgery, patients who underwent TIPS in the preoperative period had a higher hazard of postoperative mortality compared to propensity-matched controls without TIPS. Our study and comparable prior studies are limited by heterogeneous indications for TIPS, with a minority being placed specifically for preoperative risk mitigation. Despite these shared limitations, our results differ from recent studies that associate recent TIPS with reduced postoperative risk.^13,14,15^ There are several potential explanations for this. First, the VHA is male-predominant and relatively enriched with metabolic and cardiovascular comorbidities, which could impact operative risk associated with TIPS. Second, the VHA cohort comprises centers that range from community to academic-affiliated. Thus, it is possible that preoperative TIPS may mitigate risk in highly experienced centers, but when the practice is extrapolated to more diverse centers, this association does not persist. Finally, we have aimed to overcome potential limitations from prior controlled studies. Aryan et al^14^ reported reduced postoperative mortality in TIPS versus no-TIPS patients after abdominal surgery in a single-center study; however, no matching or multivariable adjustment was performed. Chang et al^15^ performed a 1:1 PSM analysis; however, the degree of matching across surgery types lacked granularity, limited to visceral versus nonvisceral and potentially leading to residual confounding. These points highlight major strengths of our study: we were able to (1) draw well-matched controls from over 13,000 patients with cirrhosis without preoperative TIPS and (2) account for demographic, comorbidity, laboratory, and more granular surgical data available through VOCAL-VASQIP. Moreover, the large pool of potential controls allowed for repeated resampling with a random number generator, which in all instances demonstrated an increased hazard of postoperative mortality associated with preoperative TIPS placement.

Assessing the evolution of liver-related labs in detail is a critical aspect of this study, as it differentiates this work from other recent publications and provides a plausible mechanism for our findings. Piecha and colleagues and Chang and colleagues reported benefits with preoperative TIPS in multivariable-adjusted and matched analyses, respectively; however, both used laboratory studies from the immediate preoperative time period. Importantly, laboratory values in the immediate preoperative period may function as mediators of risk—reflecting changes in liver function related to preoperative TIPS—rather than confounders to be adjusted. If so, adjusting or matching based on immediate preoperative labs would be expected to bias results. By contrast, our study identified patients who were clinically similar 6 months before surgery who then underwent similar surgeries with similar risk profiles, therefore enabling us to evaluate the impact of preoperative TIPS in a real-world setting where decisions surrounding TIPS are often made weeks or months in advance of surgery. We found that although labs were similar at baseline (6 mo before surgery), patients with TIPS had evidence of worsened liver synthetic function in the immediate preoperative period, possibly due to portosystemic shunting leading to relative hypoperfusion and worsened liver function. It is also plausible that hemodynamic shifts related to anesthesia and surgery may exacerbate hypoperfusion/ischemic injury disproportionately in patients with preoperative TIPS, thus leading to the progressively worsened postoperative liver synthetic function observed in this study. Overall, this suggests that the theoretical operative benefits of portal pressure reduction with TIPS may be offset by worsened liver synthetic function, which may drive an increased risk of postoperative mortality. An additional consideration is that TIPS patients had a significantly increased need for surgery due to incarcerated abdominal hernia versus no-TIPS patients, and this should be considered as a potential harm and incorporated into decision-making surrounding TIPS placement.

To further evaluate the above hypothesis, we characterized the laboratory phenotypes associated with death in the TIPS cohort versus controls. Patients who died with TIPS had greater evidence of liver synthetic dysfunction (higher bilirubin and higher MELD-Na) compared to those who died without TIPS, once again suggesting that relative hypoperfusion, likely exacerbated by anesthesia and surgery, may precipitate liver failure and death.

Finally, calculated VPS for both groups revealed generally excellent discrimination for the non-TIPS group whether computed with a 6-month baseline or immediate preoperative labs. In the TIPS group, however, discrimination was only fair when baseline labs were used but improved from very good to excellent when using immediate preoperative labs. This is likely related to the worsening liver synthetic function noted after TIPS placement, which is not captured on baseline labs. Overall, however, discrimination of VPS was inferior for the TIPS group, with a slight underestimation of surgical risk. These findings suggest that VPS should be used thoughtfully in preoperative TIPS patients and highlight the importance of repeat surgical risk stratification (eg, repeat VPS calculation) using data from the immediate preoperative period. Future cirrhosis surgical risk stratification tools should consider incorporating the placement of preoperative TIPS to improve prediction accuracy.

We acknowledge several important limitations of our study. First, there is possible confounding by indication due to heterogeneity in reported reasons for TIPS placement, including patients who underwent TIPS for indications other than presurgical optimization. As a result, these patients could represent a more vulnerable subpopulation, thus contributing to the increased mortality noted in the TIPS group. As mentioned above, other retrospective studies in this space have similar limitations with less rigorous adjustment for these key confounders.^13,15^ In our study, we accounted for key covariates in PSM that may have differentiated risk in such patients, including ascites, HE, and severity of liver disease, among others, thus mitigating this risk of bias. This is evidenced by similar liver function–related labs between TIPS and non-TIPS patients at a 6-month preoperative baseline. Ultimately, prospective trials of preoperative TIPS are needed to definitively address this confounding by indication and this is an important area of future study. Second, residual confounding is possible, although we used granular and large-scale data to achieve optimal matching. Third, as noted previously, there are potential external validity limitations related to the use of the VHA cohort. Next, since propensity matching was performed using a large sample of controls who did not receive preoperative TIPS relative to a small sample of patients who did receive TIPS, the primary PSM-based analysis results are affected by small absolute differences in measured outcomes in the TIPS group and could plausibly reflect random chance. To address this, we randomly iterated the PSM process and analyzed it 500 times to illustrate the potential distribution of HRs, each of which yielded a parameter estimate with an HR >1. Finally, misclassification of exposures is possible, however we used validated algorithms wherever possible to minimize this issue.

In conclusion, in a well-matched cohort of patients with cirrhosis undergoing major surgery, placement of TIPS within 6 months before surgery was associated with an increased hazard of postoperative mortality. While liver function–related labs were similar between groups during a 6-month baseline preoperative period, patients who underwent preoperative TIPS had worsened liver synthetic function in the immediate preoperative period that continued in the postoperative period. A potential mechanism is hypoperfusion and ischemic hepatic injury during anesthesia and surgery in a susceptible host. Overall, our findings suggest that the risks and benefits of TIPS should be carefully considered in patients who may subsequently undergo surgery; however, prospective and randomized studies of TIPS placed specifically for preoperative optimization are needed to identify specific clinical characteristics that may be associated with favorable or poor outcomes from TIPS placement before surgery.

## Supplementary Material
