# Supplementary material for: Recent TIPS increases postoperative mortality: A national cohort study
Source: Hepatol Commun. 2024 Nov 29;8(12):e0577. doi: 10.1097/HC9.0000000000000577 (PMC11608713; doi:10.1097/HC9.0000000000000577)
Supplement: Supplementary file 1 [file hc9-8-e0577-s001.docx]

**Supplemental Table 1 – Classification of Major Surgery Categories**

| **Surgery Category** | **CPT Code** |
| --- | --- |
| Spine/CNS | 22100, 22210, 22548, 22551, 22554, 22558, 22595, 22600, 22610, 22612, 22614, 22630, 22632, 22633, 22810, 22840, 22842, 22845, 61304, 61312, 61450, 61510, 61512, 61518, 61521, 61556, 61570, 61580, 61581, 61697, 61705, 62141, 62142, 63001, 63003, 63005, 63012, 63015, 63016, 63017, 63020, 63030, 63040, 63042, 63045, 63047, 63050, 63051, 63075, 63076, 63081, 63082, 63085, 63102, 63200, 63266, 63267, 63272, 63275, 63276, 63301 |
| Rectum/GU/RP | 45160, 45171, 45172, 49060, 50220, 50225, 50230, 50234, 50240, 50360, 50380, 50542, 50543, 50545, 50546, 50548, 50600, 50605, 50727, 50785, 50800, 50820, 50947, 51500, 51520, 51535, 51590, 51595, 51596, 55530, 55801, 55810, 55821, 55831, 55840, 55842, 55845, 55862, 55866, 58541, 58571, 58660, 58960, 60540, 60650 |
| Ortho | 23332, 23410, 23412, 23420, 23470, 23472, 23473, 23474, 23515, 23550, 23615, 23616, 23630, 23660, 24430, 24435, 24515, 24516, 24545, 24575, 24579, 24586, 24665, 24685, 25240, 25400, 25405, 25420, 25431, 25443, 25445, 25447, 25515, 25526, 25545, 25574, 25575, 25607, 25608, 25609, 25628, 25652, 25800, 25805, 25810, 25825, 27090, 27091, 27122, 27125, 27130, 27132, 27134, 27138, 27187, 27236, 27244, 27245, 27254, 27269, 27365, 27440, 27442, 27443, 27445, 27446, 27447, 27486, 27487, 27488, 27495, 27506, 27511, 27524, 27535, 27540, 27580, 27590, 27592, 27596, 27598, 27702, 27704, 27720, 27758, 27759, 27766, 27784, 27792, 27814, 27822, 27823, 27828, 27829, 27848, 27870, 27880, 27881, 27882, 27884, 27888, 27889, 28415, 28450, 28715, 28800, 28805, 28810 |
| Abdominal Wall | 11008, 49491, 49492, 49495, 49496, 49500, 49505, 49507, 49520, 49521, 49525, 49550, 49553, 49557, 49560, 49561, 49565, 49566, 49568, 49570, 49580, 49582, 49585, 49587, 49590, 49600, 49650, 49651, 49652, 49653, 49654, 49656, 49657, 49659, 49900, 49999 |
| Vascular | 33322, 33860, 33863, 33864, 33875, 33880, 33916, 34101, 34201, 34701, 34703, 34705, 34800, 34802, 34803, 34804, 34808, 34830, 34831, 34844, 34845, 35001, 35081, 35102, 35131, 35141, 35189, 35301, 35302, 35303, 35331, 35355, 35371, 35506, 35533, 35539, 35540, 35556, 35558, 35566, 35571, 35583, 35585, 35606, 35621, 35646, 35654, 35656, 35661, 35665, 35666, 35883, 35903 |
| Chest/Cardiac | 32096, 32097, 32100, 32110, 32120, 32140, 32220, 32225, 32320, 32480, 32482, 32484, 32491, 32500, 32503, 32505, 32506, 32507, 32650, 32651, 32652, 32655, 32656, 32657, 32660, 32661, 32662, 32663, 32666, 32667, 32668, 32669, 32674, 33025, 33030, 33031, 33250, 33251, 33400, 33405, 33406, 33410, 33411, 33412, 33420, 33422, 33426, 33427, 33430, 33464, 33496, 33501, 33504, 33505, 33510, 33511, 33512, 33517, 33518, 33519, 33521, 33530, 33533, 33534, 33535, 33572, 33641, 33945, 33975, 38746, 43107, 43112, 43314, 43328 |
| Major Abdominal | 38100, 38120, 38747, 43117, 43121, 43279, 43280, 43282, 43332, 43510, 43611, 43620, 43622, 43631, 43632, 43633, 43653, 43659, 43775, 43820, 43830, 43831, 43840, 43845, 43860, 44050, 44120, 44121, 44139, 44140, 44141, 44143, 44144, 44145, 44146, 44147, 44150, 44155, 44160, 44180, 44186, 44188, 44202, 44204, 44205, 44206, 44207, 44208, 44210, 44211, 44213, 44227, 44238, 44310, 44314, 44320, 44346, 44602, 44604, 44620, 44625, 44640, 44799, 44950, 44955, 44970, 45110, 45119, 45395, 45563, 47460, 47562, 47563, 47600, 47605, 47620, 48105, 48140, 48146, 48150, 48153, 48154, 48520, 48548, 49000, 49002, 49020, 49320, 49321, 49324, 49325, 49329, 49402 |

**Supplemental Table 2 – Patients Characteristics of Cohort Prior to Propensity Score Matching**

| **Factor** | **No Pre-Operative TIPS**  **(N=13,439)** | **Pre-Operative TIPS**  **(N=43)** | **p-value** |
| --- | --- | --- | --- |
| Age, median (IQR) | 65 (60, 69) | 63 (55, 68) | 0.080 |
| Male Sex | 13054 (97.1%) | 39 (90.7%) | 0.012 |
| Race/ethnicity |  |  | 0.037 |
| White | 8428 (62.7%) | 35 (81.4%) |  |
| Black | 2843 (21.2%) | 1 (2.3%) |  |
| Hispanic | 1034 (7.7%) | 4 (9.3%) |  |
| Asian | 159 (1.2%) | 0 (0.0%) |  |
| Other | 975 (7.3%) | 3 (7.0%) |  |
| Etiology |  |  | <0.001 |
| Hepatitis C Virus | 2901 (21.6%) | 2 (4.7%) |  |
| Hepatitis B Virus | 116 (0.9%) | 0 (0.0%) |  |
| Alcohol-related liver disease | 4281 (31.9%) | 27 (62.8%) |  |
| HCV+ALD | 3227 (24.0%) | 6 (14.0%) |  |
| MASLD | 2632 (19.6%) | 7 (16.3%) |  |
| Other | 282 (2.1%) | 1 (2.3%) |  |
| Type of Surgery |  |  | <0.001 |
| Spine/CNS | 821 (6.1%) | 1 (2.3%) |  |
| Rectum/GU/RP | 652 (4.9%) | 1 (2.3%) |  |
| Ortho | 4445 (33.1%) | 5 (11.6%) |  |
| Abdominal Wall | 2715 (20.2%) | 26 (60.5%) |  |
| Vascular | 1136 (8.5%) | 0 (0.0%) |  |
| Chest/Cardiac | 833 (6.2%) | 0 (0.0%) |  |
| Major Abdominal | 2837 (21.1%) | 10 (23.3%) |  |
| ASA Classification |  |  | 0.023 |
| 2 | 478 (3.6%) | 1 (2.3%) |  |
| 3 | 9231 (68.7%) | 22 (51.2%) |  |
| 4 | 3665 (27.3%) | 19 (44.2%) |  |
| 5 | 65 (0.5%) | 1 (2.3%) |  |
| Emergency Status | 1471 (10.9%) | 16 (37.2%) | <0.001 |
| ***Baseline Comorbidities (6 months prior to surgery)*** | | | |
| Diabetes Mellitus | 8703 (64.8%) | 25 (58.1%) | 0.36 |
| Coronary Artery Disease | 4590 (34.2%) | 8 (18.6%) | 0.032 |
| Heart Failure | 2750 (20.5%) | 4 (9.3%) | 0.070 |
| Ascites | 2094 (15.6%) | 35 (81.4%) | <0.001 |
| Hepatic encephalopathy | 866 (6.4%) | 10 (23.3%) | <0.001 |
| ***Baseline Laboratory Studies (6 months prior to surgery)*** | | | |
| MELD-Na, median (IQR) | 10 (8, 15) | 15 (10.5, 17) | <0.001 |
| Creatinine, median (IQR) | 1.0 (0.8, 1.3) | 1.0 (0.8, 1.6) | 0.70 |
| Sodium, median (IQR) | 138 (136, 140) | 137 (132, 139) | <0.001 |
| Albumin, median (IQR) | 3.8 (3.3, 4.1) | 3.1 (2.8, 3.5) | <0.001 |
| Bilirubin, median (IQR) | 0.7 (0.5, 1.1) | 1.2 (0.8, 1.7) | <0.001 |
| INR, median (IQR) | 1.1 (1.03, 1.3) | 1.2 (1.1, 1.4) | <0.001 |
| Hemoglobin, median (IQR) | 13.3 (11.6, 14.6) | 11.7 (10.2, 13.3) | <0.001 |
| Platelet count, median (IQR) | 160 (114, 214) | 152 (85, 217) | 0.14 |

**Supplemental Figure 1 – Covariate Balance Achieved through Propensity Score Matching (Primary Analysis)**

**
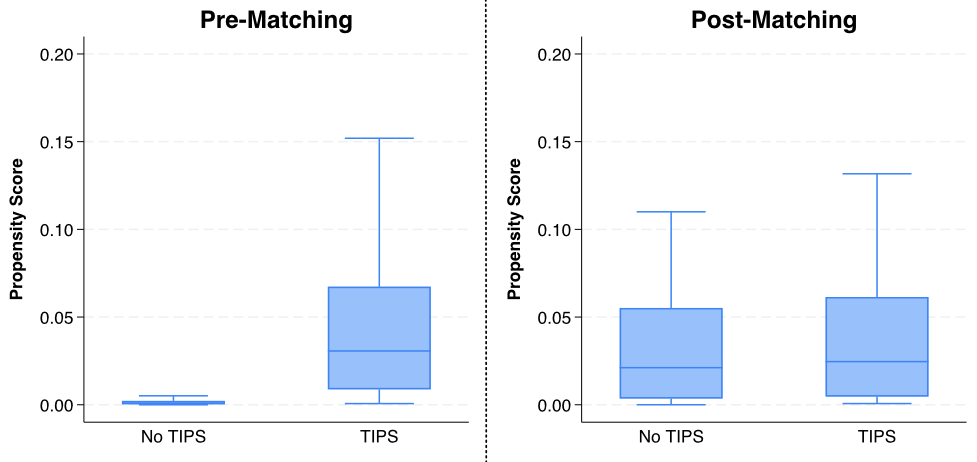
**

**Supplemental Figure 2 – Covariate Balance Achieved through Propensity Score Matching in Sensitivity Analysis Limited to Pre-Operative TIPS in the Three Months Prior to Surgery**

**
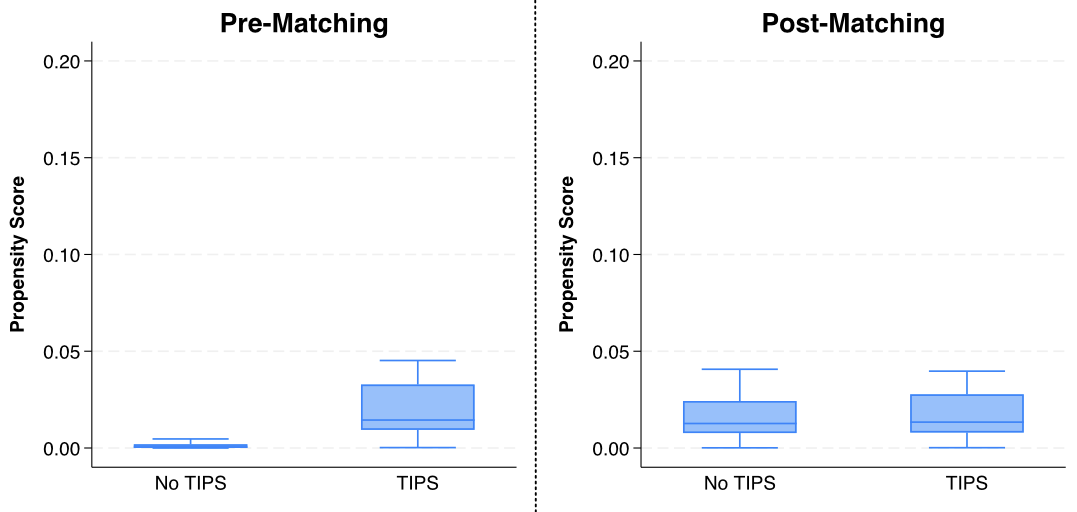
**

**Supplemental Figure 3 –Kaplan-Meier Analysis of Association between Pre-operative TIPS and Post-operative Mortality in Sensitivity Analysis Limited to Pre-Operative TIPS in the Three Months Prior to Surgery**

**
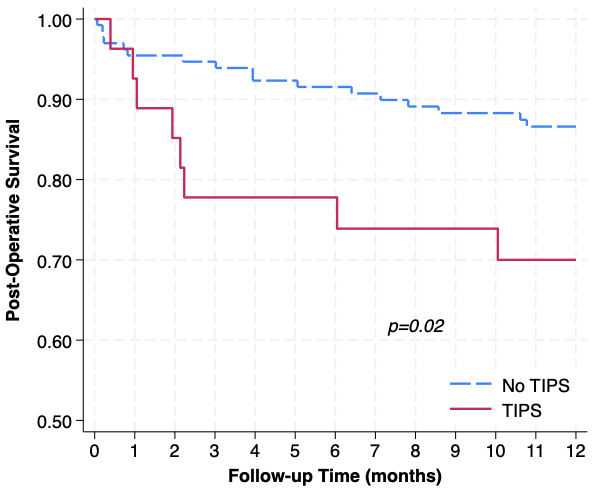
**
